# Efeito Protetor do RNA Não Codificante Longo OXCT1-AS1 na Apoptose de Células Miocárdicas Humanas Induzida pela Doxorrubicina pelo Padrão Competitivo de RNA Endógeno

**DOI:** 10.36660/abc.20230675

**Published:** 2024-06-14

**Authors:** Zhen Chen, Yijue Liu, Rui Ma, Mengli Zhang, Xian Wu, Huan Pen, Feng Gui, Yafeng Liu, Hao Xia, Niandan Hu, Bo Ai, Jun Xiong, Hongxia Xia, Wenqiang Li, Fen Ai

**Affiliations:** 1 Huazhong University of Science and Technology Tongji Medical College The Central Hospital of Wuhan Wuhan China Department of Emergency – The Central Hospital of Wuhan – Tongji Medical College – Huazhong University of Science and Technology, Wuhan – China; 2 Hubei University of Medicine Sinopharm Dongfeng General Hospital Department of Geriatric Medicine Shiyan China Department of Geriatric Medicine – Sinopharm Dongfeng General Hospital – Hubei University of Medicine, Shiyan – China; 3 Renmin Hospital of Wuhan University Department of Emergency Wuhan China Department of Emergency – Renmin Hospital of Wuhan University, Wuhan – China; 4 Renmin Hospital of Wuhan University Department of Cardiology Wuhan China Department of Cardiology – Renmin Hospital of Wuhan University, Wuhan – China

**Keywords:** RNA, Doxorrubicina, Miócitos Cardíacos, Apoptose

## Abstract

**Fundamento:**

O antibiótico quimioterápico antraciclina doxorrubicina (DOX) pode induzir cardiotoxicidade cumulativa e levar à disfunção cardíaca. RNAs não codificantes longos (lncRNAs) podem funcionar como importantes reguladores na lesão miocárdica induzida por DOX.

**Objetivo:**

Este estudo tem como objetivo investigar o papel funcional e o mecanismo molecular do RNA antisense lncRNA OXCT1 1 (OXCT1-AS1) na lesão celular miocárdica induzida por DOX in vitro.

**Métodos:**

Cardiomiócitos humanos (AC16) foram estimulados com DOX para induzir um modelo de lesão celular miocárdica. A expressão de OXCT1-AS1, miR-874-3p e BDH1 em células AC16 foi determinada por RT-qPCR. A viabilidade das células AC16 foi medida pelo ensaio XTT. A citometria de fluxo foi empregada para avaliar a apoptose de células AC16. Western blotting foi utilizado para avaliar os níveis proteicos de marcadores relacionados à apoptose. O ensaio repórter de luciferase dupla foi conduzido para verificar a capacidade de ligação entre miR-874-3p e OXCT1-AS1 e entre miR-874-3p e BDH1. O valor de p<0,05 indicou significância estatística.

**Resultados:**

A expressão de OXCT1-AS1 foi diminuída em células AC16 tratadas com DOX. A superexpressão de OXCT1-AS1 reverteu a redução da viabilidade celular e a promoção da apoptose celular causada pela DOX. OXCT1-AS1 está ligado competitivamente ao miR-874-3p para regular positivamente o BDH1. A superexpressão de BDH1 restaurou a viabilidade das células AC16 e suprimiu a apoptose celular sob estimulação com DOX. A derrubada do BDH1 reverteu a atenuação da apoptose de células AC16 mediada por OXCT1-AS1 sob tratamento com DOX.

**Conclusão:**

LncRNA OXCT1-AS1 protege células miocárdicas humanas AC16 da apoptose induzida por DOX através do eixo miR-874-3p/BDH1.

## Graphical abstract

**Figure:**
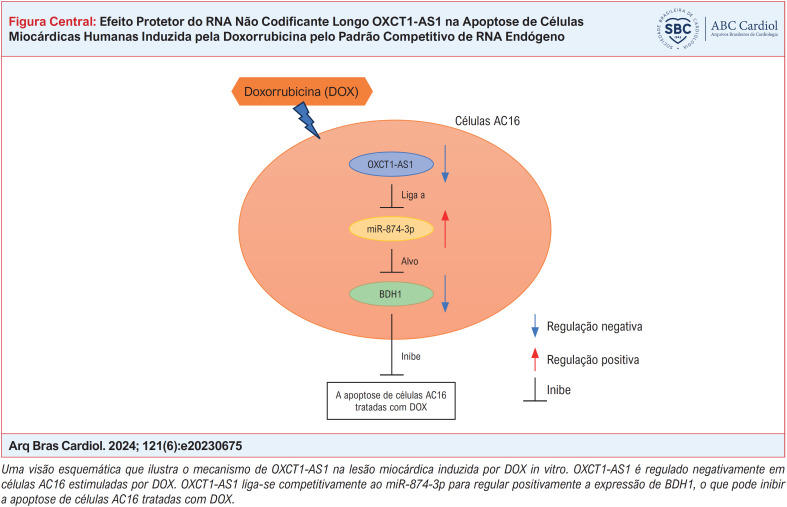

## Introdução

A insuficiência cardíaca (IC) é uma síndrome clínica complexa caracterizada por disfunção sistólica e diastólica.^1^ É o resultado final da transformação de diversas doenças cardiovasculares e está associada a altas taxas de morbidade e mortalidade.^2^ A apoptose de cardiomiócitos causa a perda de cardiomiócitos e leva à baixa contratilidade miocárdica.^3^ Evidências crescentes indicam que a apoptose de cardiomiócitos atua como um fator chave no agravamento da IC, e a supressão do remodelamento ventricular causado pela apoptose de cardiomiócitos pode melhorar o prognóstico de pacientes com IC.^4^ Assim, uma melhor compreensão do mecanismo subjacente à apoptose de cardiomiócitos na IC pode ajudar a identificar novos alvos e desenvolver estratégias mais eficazes para o tratamento da IC.

A doxorrubicina (DOX) é um antibiótico quimioterápico antraciclina que tem sido utilizado como agente antitumoral em tumores sólidos e neoplasias hematológicas.^5^ Contudo, a aplicação clínica da DOX é limitada devido à sua cardiotoxicidade cumulativa, que pode levar a um espectro de efeitos cardiotóxicos de curto e longo prazo, incluindo disfunção ventricular esquerda, cardiomiopatia e até IC.^6^ A taxa de mortalidade aumenta significativamente para 50% dentro de dois anos após a terapia com DOX.^7^ Vários mecanismos foram propostos para a cardiotoxicidade induzida pela DOX, incluindo a apoptose de cardiomiócitos.^8^ Portanto, a DOX foi utilizada para induzir um modelo de cultura celular de lesão miocárdica em nosso estudo.

RNAs não codificantes longos (lncRNAs) são um grupo de transcritos com mais de duzentos nucleotídeos que não possuem capacidade de codificação de proteínas.^9^ Estudos demonstraram que os lncRNAs participam na regulação da cardiotoxicidade induzida pela DOX na IC. Por exemplo, lncRNA NONMMUT015745 suprime a apoptose de cardiomiócitos induzida por DOX via eixo Rab2A-p53.^10^ A regulação negativa do lncRNA SOX2-OT melhora a disfunção miocárdica na IC isquêmica.^11^ LncRNA KCNQ1OT1 contribui para a apoptose de cardiomiócitos ao direcionar o FUS na IC.^12^ É importante ressaltar que um estudo anterior mencionou que a perda de RNA antisense lncRNA OXCT1 1 (OXCT1-AS1) em tecido cardíaco de engenharia humana resulta na diminuição do desenvolvimento da força contrátil. No entanto, não está claro se o OXCT1-AS1 pode afetar a toxicidade miocárdica desencadeada pela DOX.^13,14^

Evidências acumuladas demonstraram que os lncRNAs podem funcionar como RNAs endógenos concorrentes (ceRNAs) para afetar a estabilidade e tradução do mensageiro (mRNA), interagindo competitivamente com os microRNAs compartilhados (miRNAs). Vários relatórios indicaram que OXCT1-AS1 pode funcionar como um ceRNA para regular positivamente a expressão de mRNA a jusante, afetando assim os comportamentos malignos das células tumorais.^15,16^ No entanto, as moléculas a jusante envolvidas nas redes de ceRNA mediadas por OXCT1-AS1 na IC permanecem obscuras. Aqui, ferramentas de bioinformática foram utilizadas para explorar as moléculas a jusante de OXCT1-AS1, com o objetivo de compreender seu mecanismo molecular em afetar a lesão celular miocárdica induzida por DOX.

Neste estudo, nosso objetivo foi investigar o papel funcional e o mecanismo molecular do lncRNA OXCT1-AS1 em um modelo de lesão celular miocárdica induzido por DOX. Nossa hipótese é que OXCT1-AS1 poderia afetar a apoptose de cardiomiócitos induzida por DOX, modulando moléculas a jusante através da rede ceRNA.

## Materiais e métodos

### Cultura celular e tratamento

Cardiomiócitos humanos AC16 foram obtidos da ATCC (Rockville, MD, EUA) e mantidos em meio Eagle modificado por Dulbecco (Gibco, Grand Island, NY, EUA) suplementado com 10% de soro bovino fetal (Gibco) e 1% de penicilina-estreptomicina (Gibco) em atmosfera umidificada a 37°C com 95% de ar e 5% de CO2. Para induzir um modelo de lesão celular miocárdica, as células AC16 foram tratadas com 5 μM de DOX (Sangon Biotech Co., Ltd., Xangai, China) por 24 horas.^17^ Em alguns experimentos, as células AC16 foram tratadas com 5 μM de DOX por diferentes períodos (0, 0,5, 2, 6, 12, 24 e 48 h) para detecção da expressão de RNA.

### Transfecção celular

Para ensaios de superexpressão, o cDNA completo de OXCT1-AS1 e BDH1 (3-hidroxibutirato desidrogenase 1) foi amplificado e clonado no vetor pcDNA3.1 (Invitrogen, Carlsbad, CA, EUA) para construir OE-OXCT1-AS1 e OE -BDH1, com um vetor vazio (EV) como controle negativo. RNA em gancho curto direcionado a BDH1 (sh-BDH1), inibidor de miR-874-3p e o controle negativo (inibidor de NC) (Invitrogen) foram usados para ensaios de knockdown. Ao atingir 80% de confluência em placas de 6 poços, as células AC16 foram transfectadas com os vectores ou oligonucleótidos acima utilizando Lipofectamine 2000 (Invitrogen). Após 48 h de transfecção, as células AC16 foram submetidas a tratamento com DOX durante 24 h para subsequentes análises funcionais.

### Reação em cadeia da polimerase quantitativa com transcrição reversa (RT-qPCR)

O RNA total foi isolado de células AC16 tratadas utilizando o reagente TRIzol (Invitrogen). O RNA foi então transcrito reversamente em cDNA usando o kit de reagentes PrimeScript RT (TaKaRa, Dalian, China). Para a detecção da expressão OXCT1-AS1, miR-874-3p e BDH1, o qPCR em tempo real foi conduzido usando o kit SYBR Green Quantitative RT-qPCR (Sigma-Aldrich, Shanghai, China) em um CFX96 Touch Real-Time PCR Detection Sistema (Bio-Rad, Hercules, CA, EUA). A expressão gênica relativa foi calculada usando o método 2-^ΔΔCT^, com normalização para GAPDH ou U6.^18^

### Ensaio XTT

Células AC16 foram semeadas em placas de 96 poços (1×105/poço), seguidas pelos tratamentos indicados. Em seguida, as células em cada poço foram incubadas com 10 μlde solução XTT (X2000, Solarbio, Pequim, China) por mais 4 horas a 37°C. A viabilidade celular foi determinada medindo o valor da densidade óptica a 450 nm utilizando um leitor de microplacas (Bio-Rad).

### Análise de citometria de fluxo

A apoptose celular foi avaliada por citometria de fluxo utilizando o Kit de Detecção de Apoptose Anexina V-FITC (C1062M, Beyotime) de acordo com os protocolos do fabricante. Após o tratamento com DOX, as células AC16 foram colhidas, lavadas com tampão fosfato salino e depois ressuspensas em 195 μl de tampão de ligação. Posteriormente, as células foram incubadas com Anexina V-FITC (5 μl) e PI (10 μl) no escuro à temperatura ambiente durante 15 min. A apoptose celular foi medida utilizando um citômetro de fluxo (FC500MCL, Beckman Coulter, Brea, CA, EUA).

### Western blotting

A proteína total foi extraída de células AC16 utilizando tampão de lise RIPA (Solarbio). Um kit de ensaio de proteína de ácido bicinconínico (Beyotime, Xangai, China) foi utilizado para quantificar a concentração de proteína. Em seguida, quantidades iguais de amostras de proteína (20 μg) foram separadas por eletroforese em gel de poliacrilamida com dodecil sulfato de sódio a 10% e transferidas para membranas de fluoreto de polivinilideno (Beyotime). Após serem bloqueadas com leite desengordurado a 5%, as células foram incubadas a 4°C durante a noite com os seguintes anticorpos primários: anti-Bcl-2 (ab32124, 1:1000), anti-Bax (ab32503, 1:1000), anti-Cleaved caspase-3 (ab2302, 1:500), caspase-9 anti-clivada (ab2324, 1:500), anti-GAPDH (ab8245, 1:2500) (todos da Abcam, Xangai, China). Em seguida, as membranas foram lavadas três vezes com o tampão de lavagem (Beyotime), seguido de incubação com o anticorpo secundário de cabra anti-coelho conjugado com peroxidase de rábano (ab97080, 1: 5000, Abcam) à temperatura ambiente durante 1 h. Finalmente, as bandas proteicas foram visualizadas utilizando um reagente de quimiluminescência melhorado (BeyoECL Plus, Beyotime) e a intensidade relativa da banda foi analisada utilizando o software ImageJ.

### Ensaio de repórter de luciferase dupla

Locais de ligação putativos entre miR-874-3p e OXCT1-AS1, e entre miR-874-3p e BDH1 3’UTR foram previstos pelos bancos de dados ENCORI e TargetScan, respectivamente. As sequências de ligação previstas em OXCT1-AS1 e BDH1 3’UTR foram mutadas pelo QuickMutation™ Site-Directed Mutagenesis Kit (Beyotime). Posteriormente, as sequências de tipo selvagem (WT) e mutante (MUT) de OXCT1-AS1 e BDH1 3’UTR foram clonadas no vetor pmirGLO (Promega, Madison, WI, EUA) para construir OXCTA-AS1-WT/MUT e BDH1 3’ UTR-WT/MUT. Em seguida, as células AC16 foram co-transfectadas com os plasmídeos repórteres acima juntamente com o inibidor miR-874-3p ou inibidor de NC usando Lipofectamine 2000. Após 48 h, um sistema de ensaio repórter de luciferase dupla (Promega) foi usado para avaliar a atividade da luciferase, com normalização para Renilla luciferase.^19^

### Análise estatística

O software SPSS 22.0 (IBM, Armonk, NY, EUA) foi utilizado para análise dos resultados experimentais. Cada experimento foi repetido pelo menos três vezes. O tamanho da amostra foi definido por conveniência. A distribuição normal dos dados foi avaliada pelo teste de Kolmogorov-Smirnov. Todos os dados quantitativos deste estudo são apresentados como médias ± desvio padrão. As comparações entre os dois grupos foram realizadas por meio de t de Student. Quanto às comparações entre múltiplos grupos, foi aplicada análise de variância unidirecional seguida do teste de Tukey. O valor de p < 0,05 foi considerado estatisticamente significativo.

## Resultados

### A superexpressão de OXCT1-AS1 inibe a apoptose de células miocárdicas humanas desencadeada por DOX

**Figura 1:**
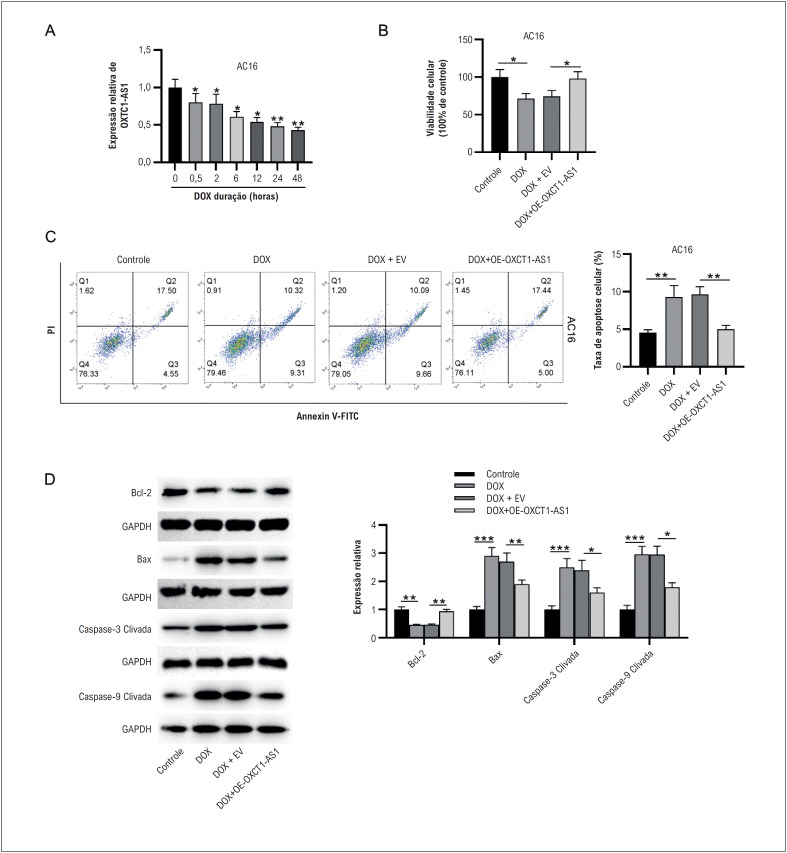
A superexpressão de OXCT1-AS1 inibe a apoptose de células miocárdicas humanas desencadeada por DOX. A) Células AC16 foram tratadas com DOX por 0,5, 2, 6, 12, 24 e 48 h, e a expressão relativa de OXCT1-AS1 em células miocárdicas humanas AC16 foi medida por RT-qPCR. B) A viabilidade das células AC16 foi determinada pelo ensaio XTT. C) A citometria de fluxo foi realizada para detectar apoptose celular. D) Os níveis de proteína dos genes relacionados à apoptose foram analisados por western blotting. n=3.*p< 0,05, **p< 0,01, ***p< 0,001. DOX: doxorrubicina; EV: vetor vazio; OE: superexpressão.

Células miocárdicas AC16 humanas foram estimuladas com DOX por 0,5, 2, 6, 12, 24 e 48 h. Comparado com o grupo controle, o nível de expressão de OXCT1-AS1 diminuiu de forma dependente do tempo após o tratamento com DOX (Figura 1A). Como mostrado pelo ensaio XTT, o tratamento com DOX prejudicou significativamente a viabilidade das células AC16, enquanto este efeito foi neutralizado pela superexpressão de OXCT1-AS1 (Figura 1B). Em seguida, avaliamos a apoptose celular via citometria de fluxo. Os resultados mostraram que a DOX promoveu a apoptose de células AC16, enquanto a superexpressão de OXCT1-AS1 reduziu a apoptose de células AC16 sob estimulação com DOX (Figura 1C). Consistentemente, western blotting revelou que o tratamento com DOX levou a um aumento nos níveis de expressão de proteínas pró-apoptóticas, incluindo Bax, caspase-3 clivada e caspase-9 clivada e uma diminuição no nível de expressão da proteína anti-apoptótica Bcl- 2 em células AC16 (Figura 1D). No entanto, os efeitos acima causados pelo tratamento com DOX foram atenuados de forma proeminente pela superexpressão de OXCT1-AS1 (Figura 1D). Coletivamente, esses achados revelaram que a superexpressão de OXCT1-AS1 poderia atenuar a apoptose de células AC16 induzida por DOX in vitro.

### OXCT1-AS1 se liga ao miR-874-3p

**Figura 2:**
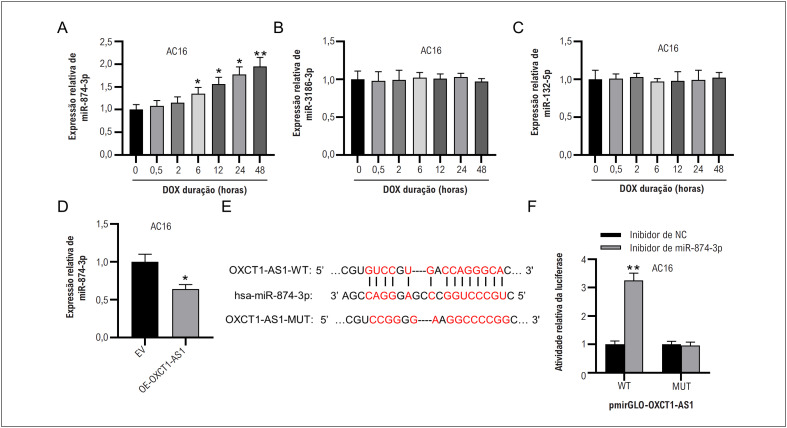
OXCT1-AS1 liga-se ao miR-874-3p. A-C) Os níveis de expressão de possíveis miRNAs a jusante de OXCT1-AS1 em AC16 tratado com DOX foram examinados por RT-qPCR. D) A expressão de miR-874-3p foi mostrada por RT-qPCR após superexpressão de OXCT1-AS1. E) O sítio de ligação entre OXCT1-AS1 e miR-874-3p foi previsto pelo banco de dados ENCORI. F) O ensaio repórter da luciferase foi conduzido para verificar a capacidade de ligação entre miR-874-3p e OXCT1-AS1 em células AC16, n=3. *p< 0,05, **p< 0,01 vs. 0 h ou EV.DOX, doxorrubicina; EV: vetor vazio; OE: superexpressão; NC: controle negativo; WT: tipo largo; MUT: mutante.

Para determinar o mecanismo potencial pelo qual OXCT1-AS1 afetou a apoptose de células AC16 sob estimulação DOX, identificamos então três miRNAs candidatos a jusante de OXCT1-AS1 através do site ENCORI. Notavelmente, o tratamento com DOX aumentou de forma dependente do tempo a expressão de miR-874-3p em células AC16 (Figura 2A), mas não teve efeito significativo na expressão de miR-3186-3p e miR-132-5p (Figura 2B-C). Assim, o miR-874-3p foi selecionado para investigação adicional. De acordo com o RT-qPCR, a expressão do miR-874-3p foi significativamente suprimida pela superexpressão de OXCT1-AS1 (Figura 2D). O sítio de ligação do miR-874-3p no OXCT1-AS1 foi previsto pelo banco de dados ENCORI (Figura 2E). Em seguida, mutamos o sítio de ligação previsto em OXCT1-AS1 e realizamos o ensaio repórter da luciferase. Como demonstrado pelos resultados, a regulação negativa do miR-874-3p diminuiu acentuadamente a atividade da luciferase de OXCT1-AS1-WT, mas não teve efeito significativo sobre a de OXCT1-AS1-MUT em células AC16 (Figura 2F), confirmando que OXCT1-AS1 poderia se ligar ao miR-874-3p em células AC16.

### miR-874-3p tem como alvo BDH1

**Figura 3:**
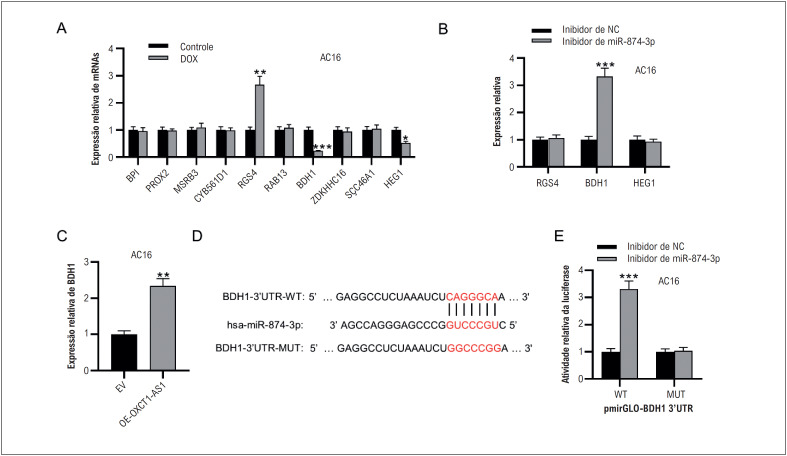
miR-874-3p tem como alvo BDH1. A)Os níveis de expressão de dez mRNAs que possuem potenciais sítios de ligação ao miR-874-3p foram avaliados por RT-qPCR. (B) RT-qPCR foi realizado para avaliar a expressão de RGS4, BDH1 e HEG1 em células AC16 com ou sem inibição de miR-874-3p. (C) RT-qPCR mostrou o nível de expressão de BDH1 após superexpressão de OXCT1-AS1. (D) O sítio de ligação entre miR-874-3p e OXCT1-AS1 foi previsto pelo TargetScan. (E). O ensaio repórter da luciferase foi realizado para validar a relação de ligação entre miR-874-3p e BDH1 em células AC16. n=3.**p< 0,01, ***p< 0,001. DOX: doxorrubicina; EV: vetor vazio; OE: superexpressão; NC: controle negativo; WT: tipo largo; MUT: mutante.

Para descobrir melhor o mecanismo de OXCT1-AS1 na lesão celular miocárdica induzida por DOX, pesquisamos os potenciais alvos a jusante do miR-874-3p usando o banco de dados TargetScan e selecionamos os dez principais genes. Detectamos então os níveis de expressão desses genes em células AC16 estimuladas com ou sem DOX. Como mostrado na Figura 3A, três dos dez genes (RGS4, BDH1, HEG1) foram significativamente expressos diferencialmente entre o grupo DOX e o grupo controle. Para confirmar ainda mais o gene alvo, foi realizado RT-qPCR para examinar os níveis de expressão de RGS4, BDH1 e HEG1 após a inibição do miR-874-3p. Em comparação com o grupo controle, apenas o BDH1 foi proeminentemente regulado positivamente no grupo depletado de miR-874-3p (Figura 3B). Além disso, a superexpressão de OXCT1-AS1 aumentou o nível de expressão de BDH1 (Figura 3C). O banco de dados TargetScan previu um potencial sítio de ligação do miR-874-3p na 3’UTR do BDH1 (Figura 3D). O ensaio repórter da luciferase foi conduzido para verificar a relação de ligação entre as duas moléculas. Em comparação com a do grupo inibidor de NC, a atividade da luciferase do BDH1 3’UTR-WT foi significativamente aumentada no grupo do inibidor miR-874-3p, mas permaneceu quase inalterada após a mutação (Figura 3E). Consequentemente, o miR-874-3p poderia ter como alvo o 3’UTR do BDH1.

### BDH1 suprime a apoptose de células miocárdicas humanas estimuladas por DOX

**Figura 4:**
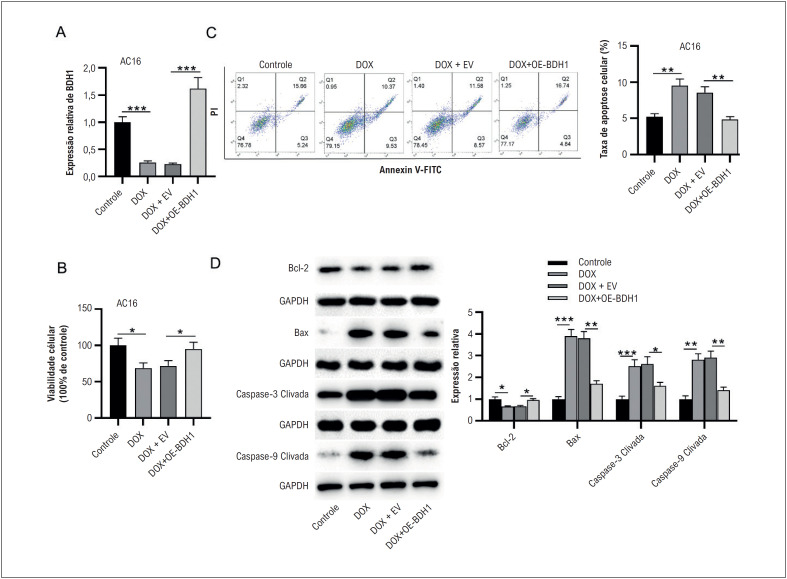
BDH1 suprime a apoptose de células miocárdicas humanas estimuladas por DOX. A) A expressão de BDH1 em células AC16 de diferentes grupos (controle, DOX, DOX-EV e DOX+OE-OXCT1-AS1) foi examinada por RT-qPCR. (B) A viabilidade das células AC16 após os tratamentos indicados foi detectada pelo ensaio XTT. (C) A apoptose das células AC16 em cada grupo foi examinada por citometria de fluxo. (D) Os efeitos da superexpressão de BDH1 nos níveis de proteína de genes relacionados à apoptose (Bax, caspase-3 clivada, caspase-9 clivada e Bcl-2) em células AC16 tratadas com DOX foram analisados por western blotting. n=3.*p< 0,05, **p< 0,01, ***p< 0,001. DOX: doxorrubicina; EV: vetor vazio; OE: superexpressão.

Para revelar o papel funcional do BDH1, superexpressamos o BDH1 em células AC16 tratadas com DOX. RT-qPCR demonstrou que a expressão de BDH1 foi significativamente regulada positivamente em células AC16 após transfecção de OE-BDH1 (Figura 4A). Como mostrado pelo ensaio XTT e citometria de fluxo, a superexpressão de BDH1 reverteu marcadamente o comprometimento da viabilidade celular AC16 induzido por DOX e a promoção da apoptose celular (Figura 4B-C). Além disso, em comparação com o grupo DOX + EV, o DOX + OE-BDH1 apresentou níveis significativamente diminuídos de Bax, caspase-3 clivada e caspase-9 clivada e níveis elevados de Bcl-2 (Figura 4D). Em conjunto, a superexpressão de BDH1 poderia aliviar a apoptose de células AC16 sob estimulação com DOX.

### Silenciamento de BDH1 resgata o efeito supressor de OXCT1-AS1 na apoptose de células AC16 sob estimulação DOX

**Figura 5:**
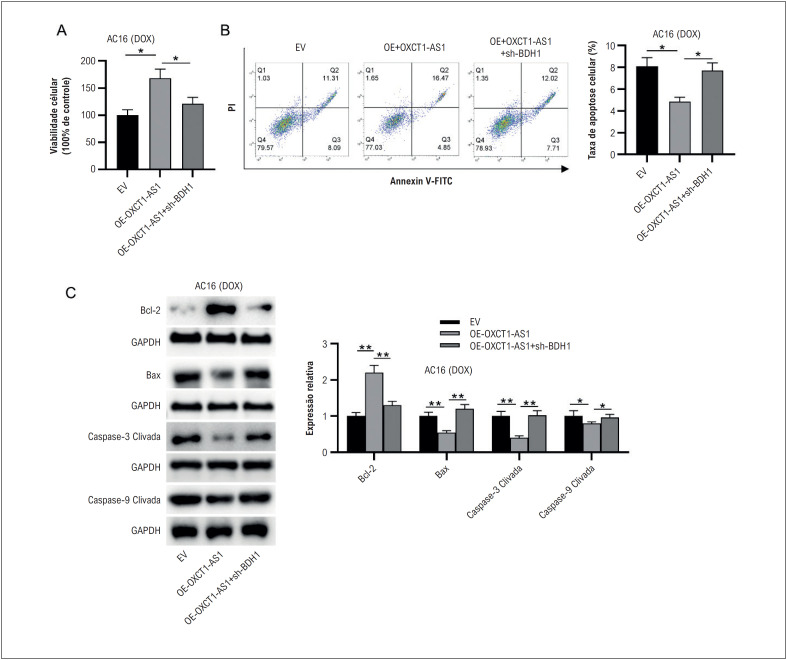
O silenciamento de BDH1 resgata o efeito supressor de OXCT1-AS1 na apoptose de células AC16 tratadas com DOX. Células AC16 foram transfectadas com EV, OE-OXCT1-AS1 ou OE-OXCT1-AS1 + sh-BDH1, seguido de estimulação com DOX. (A) A viabilidade das células AC16 foi avaliada pelo ensaio XTT. (B) A apoptose de células AC16 foi demonstrada por citometria de fluxo. (C) Western blotting foi utilizado para avaliar os níveis de proteína relacionados à apoptose em células AC16. n=3.*p< 0,05, **p< 0,01. DOX: doxorrubicina; EV: vetor vazio; OE: superexpressão; sh: RNA em gancho curto.

Posteriormente, experimentos de resgate foram conduzidos para verificar o papel do eixo OXCT1-AS1/miR-874-3p/BDH1 na lesão celular miocárdica induzida por DOX. Células AC16 foram co-transfectadas com OE-OXCT1-AS1 mais sh-BDH1. Notavelmente, o knockdown de BDH1 reverteu significativamente o aumento da viabilidade celular AC16 mediado pela superexpressão de OXCT1-AS1 e a atenuação da apoptose celular, como demonstrado pelo ensaio XTT e análise de citometria de fluxo, respectivamente (Figura 5A-B). De acordo com western blotting, o knockdown de BDH1 resgatou os níveis de proteína regulados negativamente de Bax, caspase-3 clivada e caspase-9 clivada e os níveis regulados positivamente de Bcl-2 causados pela superexpressão de OXCT1-AS1 (Figura 5C). Portanto, esses dados revelaram que o OXCT1 poderia aliviar a apoptose de cardiomiócitos AC16 induzida por DOX, regulando positivamente o BDH1.

## Discussão

O presente estudo revelou que a superexpressão de OXCT1-AS1 poderia aumentar a viabilidade dos cardiomiócitos AC16 e suprimir a apoptose dos cardiomiócitos sob estimulação com DOX. Mecanisticamente, descobrimos que o OXCT1-AS1 interagiu competitivamente com o miR-874-3p para regular positivamente a expressão de BDH1 em células AC16. Além disso, o silenciamento do BDH1 poderia reverter os efeitos acima mediados pela superexpressão de OXCT1-AS1 em células AC16 estimuladas por DOX. O mecanismo investigado neste estudo foi mostrado na Figura Central.

A cardiotoxicidade induzida pela DOX é um problema clínico crítico no manejo de diferentes tipos de malignidades.^20^ As evidências sugerem que a DOX pode provocar apoptose maciça de cardiomiócitos e resultar em disfunção cardíaca grave, levando finalmente à IC.^21^ A inibição da apoptose de cardiomiócitos pode melhorar a disfunção cardíaca induzida por DOX.^22^ Recentemente, o papel dos lncRNAs nas doenças cardiovasculares tem sido relatado em numerosos estudos.^23,24^ Além disso, a desregulação dos lncRNAs contribui para a apoptose de cardiomiócitos induzida por DOX.^10,25^ Como mencionado acima, as evidências sugerem que o lncRNA OXCT1-AS1 está associado ao desenvolvimento da força contrátil no tecido cardíaco humano e pode servir como um regulador da sobrevivência dos cardiomiócitos.^13^ No entanto, o papel preciso e o mecanismo do OXCT1-AS1 na toxicidade miocárdica induzida pela DOX ainda não foram estudados. Consistente com evidências anteriores, nosso estudo revelou que o tratamento com DOX prejudicou a viabilidade das células AC16 e induziu a apoptose celular in vitro. Além disso, o tratamento com DOX regulou negativamente a expressão de OXCT1-AS1 em cardiomiócitos AC16. Evidências sugerem que o silenciamento de OXCT1-AS1 aumenta a apoptose de cardiomiócitos humanos e de camundongos.^13^ Da mesma forma, nosso estudo mostrou que a superexpressão de OXCT1-AS1 atenuou significativamente os efeitos acima causados pelo tratamento com DOX, indicando o papel cardioprotetor de OXCT1-AS1 na lesão miocárdica induzida por DOX.

Está bem estabelecido que os lncRNAs podem se ligar competitivamente aos miRNAs e subsequentemente liberar a degradação de mRNAs mediada por miRNAs, nomeadamente a teoria do ceRNA.^26^ Para determinar o mecanismo molecular de OXCT1-AS1 na regulação da lesão celular miocárdica induzida por DOX, investigamos os miRNAs e mRNAs a jusante através de análise de bioinformática e validação experimental. Como resultado, o OXCT1-AS1 poderia se ligar ao miR-874-3p para regular positivamente o BDH1. Relatórios anteriores demonstraram o envolvimento do miR-874-3p em múltiplas doenças. Por exemplo, o miR-874-3p pode suprimir a osteoporose visando a leptina.^27^ Wei et al. propuseram que o miR-874-3p facilita a apoptose de células da granulosa induzida por testosterona através da supressão da desacetilação de p53 mediada por HDAC1.^28^ No entanto, até onde sabemos, não há estudo sobre o papel do miR-874-3p na lesão miocárdica induzida por DOX. Aqui, nosso estudo revelou a regulação positiva do miR-874-3p em células AC16 estimuladas por DOX, indicando seu papel potencial na cardiotoxicidade desencadeada por DOX. Mais investigações são necessárias para elucidar os achados.

O BDH1 está localizado no cromossomo 3q29 e pertence à família de genes da desidrogenase/redutase de cadeia curta. Relatórios anteriores indicaram que o BDH1 atua como um regulador crítico em múltiplas doenças, como doença renal diabética, doença hepática gordurosa e câncer.^29-31^ É importante ressaltar que foi revelado que a superexpressão de BDH1 atenua a apoptose de cardiomiócitos^32^ de camundongos induzida por peróxido de hidrogênio. Consistentemente, nosso estudo mostrou que o BDH1 foi significativamente regulado negativamente em células AC16 tratadas com DOX, e a superexpressão de BDH1 suprimiu a apoptose de células AC16 induzida por DOX. Além disso, o knockdown de BDH1 reverteu a atenuação mediada por OXCT1-AS1 da apoptose de cardiomiócitos humanos sob estimulação com DOX, confirmando o efeito cardioprotetor do eixo OXCT1-AS1/miR-874-3p/BDH1.

Vale ressaltar que este estudo ainda apresenta algumas limitações. Primeiro, faltavam dados in vivo neste estudo. Em segundo lugar, considerando a complexidade dos mecanismos, os reguladores a montante ou as vias de sinalização a jusante do OXCT1-AS1 precisam ser explorados em estudos futuros. Além disso, investigações adicionais podem se beneficiar da exploração dos papéis de RGS4 e HEG1 que são desregulados após a estimulação com DOX.

## Conclusões

Em conclusão, o presente estudo demonstra pela primeira vez que OXCT1-AS1 pode proteger células miocárdicas AC16 humanas da apoptose induzida por DOX, regulando o eixo miR-874-3p/BDH1. Nossos achados podem fornecer novas ideias para a atenuação da lesão miocárdica desencadeada pela DOX e a prevenção da IC.
