# Supplementary material for: Efeito Protetor do RNA Não Codificante Longo OXCT1-AS1 na Apoptose de Células Miocárdicas Humanas Induzida pela Doxorrubicina pelo Padrão Competitivo de RNA Endógeno
Source: Arq Bras Cardiol. 2024 Jun 14;121(6):e20230675. [Article in Portuguese] doi: 10.36660/abc.20230675 (PMC11216341; doi:10.36660/abc.20230675)
Supplement: Translation

# Translation: Protective Effect of Long Noncoding RNA OXCT1-AS1 on Doxorubicin-Induced Apoptosis of Human Myocardial Cells by the Competitive Endogenous RNA Pattern

**DOI:** 10.36660/abc.20230675i

**Authors:** Zhen Chen, Yijue Liu, Rui Ma, Mengli Zhang, Xian Wu, Huan Pen, Feng Gui, Yafeng Liu, Hao Xia, Niandan Hu, Bo Ai, Jun Xiong, Hongxia Xia, Wenqiang Li, Fen Ai

**Affiliations:** 1 Wuhan China Department of Emergency – The Central Hospital of Wuhan – Tongji Medical College – Huazhong University of Science and Technology, Wuhan – China; 2 Shiyan China Department of Geriatric Medicine – Sinopharm Dongfeng General Hospital – Hubei University of Medicine, Shiyan – China; 3 Wuhan China Department of Emergency – Renmin Hospital of Wuhan University, Wuhan – China; 4 Wuhan China Department of Cardiology – Renmin Hospital of Wuhan University, Wuhan – China

**Keywords:** RNA, Doxorubicin, Cardiac Myocytes, Apoptosis

## Abstract

**Background:**

The anthracycline chemotherapeutic antibiotic doxorubicin (DOX) can induce cumulative cardiotoxicity and lead to cardiac dysfunction. Long non-coding RNAs (lncRNAs) can function as important regulators in DOX-induced myocardial injury.

**Objective:**

This study aims to investigate the functional role and molecular mechanism of lncRNA OXCT1 antisense RNA 1 (OXCT1-AS1) in DOX-induced myocardial cell injury in vitro.

**Methods:**

Human cardiomyocytes (AC16) were stimulated with DOX to induce a myocardial cell injury model. OXCT1-AS1, miR-874-3p, and BDH1 expression in AC16 cells were determined by RT-qPCR. AC16 cell viability was measured by XTT assay. Flow cytometry was employed to assess the apoptosis of AC16 cells. Western blotting was used to evaluate protein levels of apoptosis-related markers. Dual-luciferase reporter assay was conducted to verify the binding ability between miR-874-3p and OXCT1-AS1 and between miR-874-3p and BDH1. The value of p<0.05 indicated statistical significance.

**Results:**

OXCT1-AS1 expression was decreased in DOX-treated AC16 cells. Overexpression of OXCT1-AS1 reversed the reduction of cell viability and promotion of cell apoptosis caused by DOX. OXCT1-AS1 is competitively bound to miR-874-3p to upregulate BDH1. BDH1 overexpression restored AC16 cell viability and suppressed cell apoptosis under DOX stimulation. Knocking down BDH1 reversed OXCT1-AS1-mediated attenuation of AC16 cell apoptosis under DOX treatment.

**Conclusion:**

LncRNA OXCT1-AS1 protects human myocardial cells AC16 from DOX-induced apoptosis via the miR-874-3p/BDH1 axis.

## Introduction

Heart failure (HF) is a complex clinical syndrome characterized by systolic and diastolic dysfunction.^1^ It is the ultimate transformation result of various cardiovascular diseases and is associated with high morbidity and mortality rates.^2^ Cardiomyocyte apoptosis causes the loss of cardiomyocytes and leads to poor myocardial contractility.^3^ Mounting evidence has indicated that cardiomyocyte apoptosis acts as a key factor in the aggravation of HF, and suppression of ventricular remodeling caused by cardiomyocyte apoptosis can improve the prognosis of patients with HF.^4^ Thus, a better understanding of the underlying mechanism of cardiomyocyte apoptosis in HF may help to identify new targets and develop more effective strategies for HF treatment.

Doxorubicin (DOX) is an anthracycline chemotherapeutic antibiotic that has been used as an anti-tumor agent in solid tumors and hematologic neoplasia.^5^ However, the clinical application of DOX is limited due to its cumulative cardiotoxicity, which can lead to a spectrum of short- and long-term cardiotoxic effects, including left ventricular dysfunction, cardiomyopathy, and even HF.^6^ The mortality rate increases significantly to 50% within two years following DOX therapy.^7^ Several mechanisms have been proposed for DOX-induced cardiotoxicity, including cardiomyocyte apoptosis.^8^ Therefore, DOX was used to induce a cell culture model of myocardial injury in our study.

Long non-coding RNAs (lncRNAs) are a group of transcripts longer than two hundred nucleotides that have no protein-coding ability.^9^ Studies have shown that lncRNAs participate in regulating DOX-induced cardiotoxicity in HF. For example, lncRNA NONMMUT015745 suppresses DOX-induced cardiomyocyte apoptosis via Rab2A-p53 axis.^10^ Down-regulation of lncRNA SOX2-OT improves myocardial dysfunction in ischemic heart failure.^11^ LncRNA KCNQ1OT1 contributes to cardiomyocyte apoptosis by targeting FUS in HF.^12^ Importantly, a previous study has mentioned that loss of lncRNA OXCT1 antisense RNA 1 (OXCT1-AS1) in human-engineered heart tissue results in decreased contractile force development. However, it is unclear whether OXCT1-AS1 can affect DOX-triggered myocardial toxicity.^13,14^

Accumulating evidence has demonstrated that lncRNAs can function as competing endogenous RNAs (ceRNAs) to affect messenger (mRNA) stability and translation by competitively interacting with the shared microRNAs (miRNAs). Several reports have indicated that OXCT1-AS1 can work as a ceRNA to indirectly upregulate downstream mRNA expression, thereby affecting the malignant behaviors of tumor cells.^15,16^ However, the downstream molecules involved in OXCT1-AS1-mediated ceRNA networks in HF remain unclear. Herein, bioinformatics tools were used to explore the downstream molecules of OXCT1-AS1, aiming to understand its molecular mechanism in affecting DOX-induced myocardial cell injury.

In this study, we aimed to investigate the functional role and molecular mechanism of lncRNA OXCT1-AS1 in a myocardial cell injury model induced by DOX. We hypothesized that OXCT1-AS1 could affect DOX-induced cardiomyocyte apoptosis by modulating downstream molecules via the ceRNA network.

## Materials and methods

### Cell culture and treatment

Human AC16 cardiomyocytes were obtained from ATCC (Rockville, MD, USA) and maintained in Dulbecco's modified Eagle medium (Gibco, Grand Island, NY, USA) supplemented with 10% fetal bovine serum (Gibco) and 1% penicillin-streptomycin (Gibco) in a humidified atmosphere at 37°C with 95% air and 5% CO_2_. To induce a myocardial cell injury model, AC16 cells were treated with 5 μM DOX (Sangon Biotech Co., Ltd., Shanghai, China) for 24 h.^17^ In some experiments, AC16 cells were treated with 5 μM DOX for different periods (0, 0.5, 2, 6, 12, 24, and 48 h) for the detection of RNA expression.

### Cell transfection

For overexpression assays, the full-length cDNA of OXCT1-AS1 and BDH1 (3-hydroxybutyrate dehydrogenase 1) were amplified and cloned into the pcDNA3.1 vector (Invitrogen, Carlsbad, CA, USA) to construct OE-OXCT1-AS1 and OE-BDH1, with an empty vector (EV) as the negative control. Short hairpin RNA targeting BDH1 (sh-BDH1), miR-874-3p inhibitor, and the negative control (NC inhibitor) (Invitrogen) were used for knockdown assays. When reaching 80% confluence in 6-well plates, AC16 cells were transfected with the above vectors or oligonucleotides using Lipofectamine 2000 (Invitrogen). After 48 h of transfection, AC16 cells were subjected to DOX treatment for 24 h for subsequent functional analyses.

### Reverse transcriptase-quantitative real time PCR (RT-qPCR)

Total RNA was isolated from treated AC16 cells using TRIzol reagent (Invitrogen). RNA was then reverse transcribed into cDNA using the PrimeScript RT reagent Kit (TaKaRa, Dalian, China). For the detection of OXCT1-AS1, miR-874-3p, and BDH1 expression, real-time qPCR was conducted using SYBR Green Quantitative RT-qPCR Kit (Sigma-Aldrich, Shanghai, China) on a CFX96 Touch Real-Time PCR Detection System (Bio-Rad, Hercules, CA, USA). The relative gene expression was calculated using the 2^-ΔΔCt^ method, with normalization to GAPDH or U6.^18^

### XTT assay

AC16 cells were seeded in 96-well plates (1×10^5^/well), followed by indicated treatments. Then, cells in each well were incubated with 10 μl of XTT solution (X2000, Solarbio, Beijing, China) for an additional 4 h at 37°C. Cell viability was determined by measuring the optical density value at 450 nm using a microplate reader (Bio-Rad).

### Flow cytometry analysis

Cell apoptosis was evaluated by flow cytometry using Annexin V-FITC Apoptosis Detection Kit (C1062M, Beyotime) as per the manufacturer's protocols. After DOX treatment, AC16 cells were harvested, washed with phosphate buffer saline, and then resuspended in 195 μl binding buffer. Afterward, cells were incubated with Annexin V-FITC (5 μl) and PI (10 μl) in the dark at room temperature for 15 min. Cell apoptosis was measured using a flow cytometer (FC500MCL, Beckman Coulter, Brea, CA, USA).

### Western blotting

Total protein was extracted from AC16 cells using RIPA lysis buffer (Solarbio). A bicinchoninic acid protein assay kit (Beyotime, Shanghai, China) was used to quantify the protein concentration. Then, equal amounts of protein samples (20 μg) were separated by 10% sodium dodecyl sulfate-polyacrylamide gel electrophoresis and transferred onto polyvinylidene fluoride membranes (Beyotime). After being blocked with 5% defatted milk, cells were incubated at 4°C overnight with the following primary antibodies: anti-Bcl-2 (ab32124, 1:1000), anti-Bax (ab32503, 1:1000), anti-Cleaved caspase-3 (ab2302, 1:500), anti-Cleaved caspase-9 (ab2324, 1:500), anti-GAPDH (ab8245, 1:2500) (all from Abcam, Shanghai, China). Then, the membranes were washed three times with the wash buffer (Beyotime), followed by incubation with the horseradish peroxidase-conjugated goat anti-rabbit secondary antibody (ab97080, 1:5000, Abcam) at room temperature for 1h. Finally, protein bands were visualized using an enhanced chemiluminescence reagent (BeyoECL Plus, Beyotime) and relative band intensity was analyzed using ImageJ software.

### Dual-luciferase reporter assay

Putative binding sites between miR-874-3p and OXCT1-AS1, and between miR-874-3p and BDH1 3’UTR were predicted by ENCORI and TargetScan databases, respectively. The predicted binding sequences on OXCT1-AS1 and BDH1 3’UTR were mutated by QuickMutation™ Site-Directed Mutagenesis Kit (Beyotime). Subsequently, the wild type (WT) and mutant (MUT) sequences of OXCT1-AS1 and BDH1 3’UTR were cloned into pmirGLO vector (Promega, Madison, WI, USA) to construct OXCTA-AS1-WT/MUT and BDH1 3’UTR-WT/MUT. Then, AC16 cells were co-transfected with the above reporter plasmids together with miR-874-3p inhibitor or NC inhibitor using Lipofectamine 2000. After 48h, a dual-luciferase reporter assay system (Promega) was used to assess the luciferase activity, with normalization to Renilla luciferase.^19^

### Statistical analysis

SPSS 22.0 software (IBM, Armonk, NY, USA) was used for analyzing the experimental results. Each experiment was repeated at least three times. The sample size was defined by convenience. Normal distribution of data was assessed with Kolmogorov-Smirnov test. All quantitative data in this study are presented as means ± standard deviation. Comparisons between the two groups were performed using paired Student's *t*-tests. As for the comparisons among multiple groups, a one-way analysis of variance followed by Tukey's test was applied. The value of p < 0.05 was considered statistically significant.

## Results

### Overexpression of OXCT1-AS1 inhibits DOX-triggered apoptosis of human myocardial cells

Human AC16 myocardial cells were stimulated with DOX for 0.5, 2, 6, 12, 24, and 48 h. Compared with that in the control group, the expression level of OXCT1-AS1 was time-dependently decreased after DOX treatment (Figure 1A). As shown by the XTT assay, DOX treatment significantly impaired the viability of AC16 cells, while this effect was counteracted by OXCT1-AS1 overexpression (Figure 1B). We next assessed cell apoptosis via flow cytometry. The results showed that DOX promoted AC16 cell apoptosis, whereas OXCT1-AS1 overexpression reduced the apoptosis of AC16 cells under DOX stimulation (Figure 1C). Consistently, western blotting revealed that DOX treatment led to an increase in the expression levels of pro-apoptotic proteins, including Bax, Cleaved caspase-3, and Cleaved caspase-9 and a decrease in the expression level of the anti-apoptotic protein Bcl-2 in AC16 cells (Figure 1D). However, the above effects caused by DOX treatment were prominently abated by overexpression of OXCT1-AS1 (Figure 1D). Collectively, these findings revealed that overexpression of OXCT1-AS1 could attenuate DOX-induced apoptosis of AC16 cells *in vitro*.

**Figure 1:** Overexpression of OXCT1-AS1 inhibits DOX-triggered apoptosis of human myocardial cells. A) AC16 cells were treated with DOX for 0.5, 2, 6, 12, 24, and 48 h, and relative expression of OXCT1-AS1 in human myocardial cells AC16 was measured by RT-qPCR. B) The viability of AC16 cells was determined by XTT assay. C) Flow cytometry was conducted to detect cell apoptosis. D) Protein levels of apoptosis-related genes were analyzed by western blotting. n=3. *p< 0.05, **p< 0.01, ***p< 0.001. DOX: doxorubicin; EV: empty vector; OE: overexpression.

### OXCT1-AS1 binds to miR-874-3p

To determine the potential mechanism by which OXCT1-AS1 affected AC16 cell apoptosis under DOX stimulation, we then identified three candidate downstream miRNAs of OXCT1-AS1 via the ENCORI website. Notably, DOX treatment time-dependently increased miR-874-3p expression in AC16 cells (Figure 2A) but had no significant effect on the expression of miR-3186-3p and miR-132-5p (Figure 2B-C). Thus, miR-874-3p was selected for further investigation. According to RT-qPCR, miR-874-3p expression was significantly suppressed by overexpression of OXCT1-AS1 (Figure 2D). The binding site of miR-874-3p on OXCT1-AS1 was predicted by the ENCORI database (Figure 2E). Then, we mutated the predicted binding site on OXCT1-AS1 and performed the luciferase reporter assay. As demonstrated by the results, downregulating miR-874-3p markedly increased the luciferase activity of OXCT1-AS1-WT but had no significant effect on that of OXCT1-AS1-MUT in AC16 cells (Figure 2F), confirming that OXCT1-AS1 could bind to miR-874-3p in AC16 cells.

**Figure 2:** OXCT1-AS1 binds to miR-874-3p. A-C) Expression levels of possible downstream miRNAs of OXCT1-AS1 in DOX-treated AC16 were examined by RT-qPCR. D) miR-874-3p expression was shown by RT-qPCR after overexpressing OXCT1-AS1. E) The binding site between OXCT1-AS1 and miR-874-3p was predicted by the ENCORI database. F) Luciferase reporter assay was conducted to verify the binding ability between miR-874-3p and OXCT1-AS1 in AC16 cells. n=3. *p< 0.05, **p< 0.01 vs. 0 h or EV. DOX, doxorubicin; EV, empty vector; OE, overexpression; NC, negative control; WT, wide type; MUT, mutant.

### miR-874-3p targets BDH1

To further figure out the mechanism of OXCT1-AS1 in DOX-induced myocardial cell injury, we searched the potential downstream targets of miR-874-3p using the TargetScan database and selected the top ten genes. We then detected the expression levels of these genes in AC16 cells stimulated with or without DOX. As shown in Figure 3A, three of the ten genes (RGS4, BDH1, HEG1) were significantly differentially expressed between the DOX group and the control group. To further confirm the target gene, RT-qPCR was conducted to examine the expression levels of RGS4, BDH1, and HEG1 after miR-874-3p inhibition. In comparison to the control group, only BDH1 was prominently upregulated in the miR-874-3p-depleted group (Figure 3B). Furthermore, overexpression of OXCT1-AS1 enhanced the expression level of BDH1 (Figure 3C). TargetScan database predicted a potential binding site of miR-874-3p on the 3’UTR of BDH1 (Figure 3D). Luciferase reporter assay was conducted to verify the binding relation between the two molecules. Compared with that in the NC inhibitor group, the luciferase activity of BDH1 3’UTR-WT was significantly increased in the miR-874-3p inhibitor group but was almost unchanged after mutation (Figure 3E). Accordingly, miR-874-3p could target the 3’UTR of BDH1.

**Figure 3:** miR-874-3p targets BDH1. A) The expression levels of ten mRNAs that have potential binding sites to miR-874-3p were evaluated by RT-qPCR. (B) RT-qPCR was performed to evaluate the expression of RGS4, BDH1, and HEG1 in AC16 cells with or without miR-874-3p inhibition. (C) RT-qPCR showed the expression level of BDH1 after OXCT1-AS1 overexpression. (D) The binding site between miR-874-3p and OXCT1-AS1 was predicted by TargetScan. (E). Luciferase reporter assay was performed to validate the binding relation between miR-874-3p and BDH1 in AC16 cells. n=3. **p< 0.01, ***p< 0.001. DOX, doxorubicin; EV, empty vector; OE, overexpression; NC, negative control. WT, wide type; MUT, mutant.

### BDH1 suppresses the apoptosis of DOX-stimulated human myocardial cells

To reveal the functional role of BDH1, we overexpressed BDH1 in DOX-treated AC16 cells. RT-qPCR demonstrated that BDH1 expression was significantly upregulated in AC16 cells after transfection of OE-BDH1 (Figure 4A). As shown by XTT assay and flow cytometry, overexpressing BDH1 markedly reversed DOX-induced impairment of AC16 cell viability and promotion of cell apoptosis (Figure 4B-4C). Moreover, in comparison to the DOX + EV group, the DOX + OE-BDH1 displayed significantly decreased levels of Bax, Cleaved caspase-3, and Cleaved caspase-9 and elevated levels of Bcl-2 (Figure 4D). Taken together, overexpressing BDH1 could alleviate the apoptosis of AC16 cells under DOX stimulation.

**Figure 4:** BDH1 suppresses the apoptosis of DOX-stimulated human myocardial cells. A) BDH1 expression in AC16 cells of different groups (control, DOX, DOX-EV, and DOX+OE-OXCT1-AS1) was examined by RT-qPCR. B) The viability of AC16 cells after indicated treatments was detected by XTT assay. C) The apoptosis of AC16 cells in each group was examined by flow cytometry. D) Effects of BDH1 overexpression on the protein levels of apoptosis-related genes (Bax, Cleaved caspase-3, Cleaved caspase-9, and Bcl-2) in DOX-treated AC16 cells were analyzed by western blotting. n=3. *p< 0.05, **p< 0.01, ***p< 0.001. DOX, doxorubicin; EV, empty vector; OE, overexpression.

### Silencing of BDH1 rescues the suppressive effect of OXCT1-AS1 on AC16 cell apoptosis under DOX stimulation

Subsequently, rescue experiments were conducted to verify the role of OXCT1-AS1/miR-874-3p/BDH1 axis in DOX-induced myocardial cell injury. AC16 cells were co-transfected with OE-OXCT1-AS1 plus sh-BDH1. Notably, BDH1 knockdown significantly reversed OXCT1-AS1 overexpression-mediated enhancement of AC16 cell viability and attenuation of cell apoptosis, as demonstrated by XTT assay and flow cytometry analysis, respectively (Figure 5A-5B). According to western blotting, BDH1 knockdown rescued the downregulated protein levels of Bax, Cleaved caspase-3, and Cleaved caspase-9 and the upregulated levels of Bcl-2 caused by OXCT1-AS1 overexpression (Figure 5C). Therefore, these data revealed that OXCT1 could alleviate DOX-induced apoptosis of AC16 cardiomyocytes by upregulating BDH1.

**Figure 5:** Silencing of BDH1 rescues the suppressive effect of OXCT1-AS1 on the apoptosis of DOX-treated AC16 cells. AC16 cells were transfected with EV, OE-OXCT1-AS1, or OE-OXCT1-AS1 + sh-BDH1, followed by DOX stimulation. A) The viability of AC16 cells was evaluated by XTT assay. B) AC16 cells apoptosis was shown by flow cytometry. C) Western blotting was used to assess apoptosis-related protein levels in AC16 cells. n=3. *p< 0.05, **p< 0.01. DOX, doxorubicin; EV: empty vector; OE: overexpression; sh: short hairpin RNA.

## Discussion

The present study revealed that overexpressing OXCT1-AS1 could enhance AC16 cardiomyocyte viability and suppress cardiomyocyte apoptosis under DOX stimulation. Mechanistically, we found that OXCT1-AS1 competitively interacted with miR-874-3p to upregulate BDH1 expression in AC16 cells. Moreover, silencing of BDH1 could reverse the above effects mediated by OXCT1-AS1 overexpression on DOX-stimulated AC16 cells. The mechanism investigated in this study was shown in the Central Illustration.

DOX-induced cardiotoxicity is a critical clinical issue in managing different types of malignancies.^20^ Evidence suggests that DOX can elicit massive cardiomyocyte apoptosis and result in severe cardiac dysfunction, ultimately leading to HF.^21^ Inhibition of cardiomyocyte apoptosis can ameliorate DOX-induced cardiac dysfunction.^22^ Recently, the role of lncRNAs in cardiovascular diseases has been reported in numerous studies.^23,24^ Furthermore, dysregulation of lncRNAs contributes to DOX-induced cardiomyocyte apoptosis.^10,25^ As mentioned above, evidence suggests that lncRNA OXCT1-AS1 is associated with contractile force development in human heart tissue and may serve as a regulator of cardiomyocyte survival.^13^ However, the precise role and mechanism of OXCT1-AS1 in DOX-induced myocardial toxicity have not been studied yet. Consistent with previous evidence, our study revealed that DOX treatment impaired AC16 cell viability and induced cell apoptosis *in vitro*. Moreover, DOX treatment time-dependently downregulated OXCT1-AS1 expression in AC16 cardiomyocytes. Evidence suggests that silencing of OXCT1-AS1 enhances human and mouse cardiomyocyte apoptosis.^13^ Similarly, our study depicted that overexpressing OXCT1-AS1 significantly attenuated the above effects caused by DOX treatment, indicating the cardioprotective role of OXCT1-AS1 in DOX-induced myocardial injury.

It is well-established that lncRNAs can bind competitively to miRNAs and subsequently liberate the degradation of mRNAs mediated by miRNAs, namely ceRNA theory.^26^ To determine the molecular mechanism of OXCT1-AS1 in regulating DOX-induced myocardial cell injury, we investigated the downstream miRNAs and mRNAs via bioinformatics analysis and experimental validation. As a result, OXCT1-AS1 could bind to miR-874-3p to upregulate BDH1. Previous reports have demonstrated the involvement of miR-874-3p in multiple diseases. For example, miR-874-3pcan suppress osteoporosis by targeting leptin.^27^ Wei et al. proposed that miR-874-3p facilitates testosterone-induced apoptosis of granulosa cells via the suppression of HDAC1-mediated p53 deacetylation.^28^ However, to our knowledge, there is no study on the role of miR-874-3p in DOX-induced myocardial injury. Here, our study revealed the upregulation of miR-874-3p in DOX-stimulated AC16 cells, indicating its potential role in DOX-triggered cardiotoxicity. Further investigations are needed to elucidate the findings.

BDH1 is located at chromosome 3q29 and belongs to the short-chain dehydrogenase/reductase gene family. Previous reports have indicated that BDH1 acts as a critical regulator in multiple diseases, such as diabetic kidney disease, fatty liver disease, and cancer.^29-31^ Importantly, it has been revealed that BDH1 overexpression attenuates hydrogen peroxide-induced apoptosis of mouse cardiomyocytes.^32^ Consistently, our study depicted that BDH1 was significantly downregulated in DOX-treated AC16 cells, and overexpressing BDH1 suppressed DOX-induced AC16 cell apoptosis. In addition, BDH1 knockdown reversed OXCT1-AS1-mediated attenuation of human cardiomyocyte apoptosis under DOX stimulation, confirming the cardioprotective effect of the OXCT1-AS1/miR-874-3p/BDH1 axis.

It is worth noting that this study still has some limitations. First, *in vivo* data were lacking in this study. Secondly, considering the complexity of mechanisms, the upstream regulators or downstream signaling pathways of OXCT1-AS1 need to be explored in future studies. In addition, further investigations may benefit from exploring the roles of RGS4 and HEG1 which are dysregulated after DOX stimulation.

## Conclusions

In conclusion, the present study demonstrates for the first time that OXCT1-AS1 can protect human AC16 myocardial cells from DOX-induced apoptosis by regulating the miR-874-3p/BDH1 axis. Our findings may provide new ideas for the attenuation of DOX-triggered myocardial injury and the prevention of HF.
